# SIRT3 Overexpression Ameliorates Asbestos-Induced Pulmonary Fibrosis, mt-DNA Damage, and Lung Fibrogenic Monocyte Recruitment

**DOI:** 10.3390/ijms22136856

**Published:** 2021-06-25

**Authors:** Paul Cheresh, Seok-Jo Kim, Renea Jablonski, Satoshi Watanabe, Ziyan Lu, Monica Chi, Kathryn A. Helmin, David Gius, G. R. Scott Budinger, David W. Kamp

**Affiliations:** 1 Jesse Brown VA Medical Center, Division of Pulmonary & Critical Care Medicine, Chicago, IL 60612, USA; p-cheresh@northwestern.edu (P.C.); seokjok@med.umich.edu (S.-J.K.); ziyan@northwestern.edu (Z.L.); s-buding@northwestern.edu (G.R.S.B.); 2 Department of Medicine, Feinberg School of Medicine, Pulmonary and Critical Care Medicine, Northwestern University, Simpson & Querrey Biomedical Research Center 5-303, 303 E Superior St., Chicago, IL 60611, USA; swatanabe@northwestern.edu (S.W.); Mon.chi930@gmail.com (M.C.); helmin@northwestern.edu (K.A.H.); 3 Section of Pulmonary and Critical Care, Pritzker School of Medicine, The University of Chicago, Chicago, IL 60637, USA; reneaj@medicine.bsd.uchicago.edu; 4 Department of Radiation Oncology, Feinberg School of Medicine, Northwestern University, Chicago, IL 60611, USA; david.gius@northwestern.edu

**Keywords:** SIRT3, mtDNA damage, oxidative stress, alveolar epithelial cell, monocytes, pulmonary fibrosis

## Abstract

Alveolar epithelial cell (AEC) mitochondrial (mt) DNA damage and fibrotic monocyte-derived alveolar macrophages (Mo-AMs) are implicated in the pathobiology of pulmonary fibrosis. We showed that sirtuin 3 (SIRT3), a mitochondrial protein regulating cell fate and aging, is deficient in the AECs of idiopathic pulmonary fibrosis (IPF) patients and that asbestos- and bleomycin-induced lung fibrosis is augmented in Sirt3 knockout (*Sirt3*^−/−^) mice associated with AEC mtDNA damage and intrinsic apoptosis. We determined whether whole body transgenic SIRT3 overexpression (*Sirt3^Tg^*) protects mice from asbestos-induced pulmonary fibrosis by mitigating lung mtDNA damage and Mo-AM recruitment. Crocidolite asbestos (100 µg/50 µL) or control was instilled intratracheally in *C57Bl6* (Wild-Type) mice or *Sirt3^Tg^* mice, and at 21 d lung fibrosis (histology, fibrosis score, Sircol assay) and lung Mo-AMs (flow cytometry) were assessed. Compared to controls, *Sirt3^Tg^* mice were protected from asbestos-induced pulmonary fibrosis and had diminished lung mtDNA damage and Mo-AM recruitment. Further, pharmacologic SIRT3 inducers (i.e., resveratrol, viniferin, and honokiol) each diminish oxidant-induced AEC mtDNA damage in vitro and, in the case of honokiol, protection occurs in a SIRT3-dependent manner. We reason that SIRT3 preservation of AEC mtDNA is a novel therapeutic focus for managing patients with IPF and other types of pulmonary fibrosis.

## 1. Introduction

Idiopathic pulmonary fibrosis (IPF) is a chronic, relentlessly progressive, age-associated pulmonary disease with a median survival of only 3–5 years [1,2]. Although two FDA-approved drugs (pirfenidone and nintedanib) can slow lung function deterioration in patients with IPF and other fibrotic lung diseases, neither agent improves pulmonary function, suggesting that there is an important need for identifying novel cellular and molecular targets that can diminish pulmonary fibrosis and promote lung repair.

In patients with IPF and asbestosis (lung fibrosis following asbestos exposure) “exaggerated” lung aging is prominently implicated in the pathobiology as all nine hallmarks of the “aging phenotype” are present in fibrotic lungs including (i) genomic instability, (ii) telomere shortening, (iii) epigenetic alterations, (iv) abnormal proteostasis, (v) dysregulated nutrient sensing, (vi) mitochondrial dysfunction, (vii) altered intercellular communication, (viii) cellular senescence/apoptosis, and (ix) stem cell depletion [3,4,5,6,7,8,9]. Our group and others have focused on the mitochondria because they are prominently involved in mediating the age-associated changes occurring in patients with IPF and murine models of lung fibrosis, including bleomycin and asbestos, because they are both the source and target of reactive oxygen species (ROS) that can promote mitochondrial DNA (mtDNA) damage and intrinsic apoptosis [1,5,6,7,8,9,10,11,12,13,14,15,16,17,18]. We established that mitochondrial 8-oxoguanine DNA glycosylase 1 (mtOGG1), an mtDNA base excision repair (BER) enzyme, mitigates bleomycin- and asbestos-induced lung fibrosis by reducing AEC mtDNA damage and apoptosis [10,19,20,21]. We also reported that mitochondrial catalase enforced-expression (MCAT) transgenic mice are protected against asbestos- and bleomycin-induced lung fibrosis due in part to diminished AEC mitochondrial reactive oxygen species (mtROS) production, mtDNA damage, and apoptosis [12]. Notably, the levels of mtDNA present in the blood or bronchoalveolar lavage fluid (BALF) in patients with IPF directly correlate with mortality [22,23]. The precise source of circulating mtDNA in patients with IPF is not established, but possibilities include transforming growth factor-beta (TGFβ)-exposed lung fibroblasts or apoptotic AECs [22,23].

Sirtuin 3 (SIRT3), a mitochondrial member of the sirtuin family of NAD-dependent deacetylases, can direct cell fate and aging. Although the cellular and molecular mechanisms involved in mediating SIRT3’s function are incompletely understood, SIRT3 has beneficial effects on most members of the mitochondrial electron transport chain, the tricarboxylic acid cycle, mtOGG1, manganese superoxide dismutase (MnSOD), and mtDNA [24,25,26,27,28,29,30,31,32,33]. Accumulating evidence firmly implicates SIRT3 deficiency in the development of pulmonary fibrosis. First, SIRT3 function is reduced in the AECs and fibroblasts of patients with IPF as well as the lungs in aged mice [32,34,35]. Additionally, extracellular vesicles (EVs) from IPF lung fibroblasts contain miR-23b-3p and miR-494-3p that can reduce lung epithelial cell *SIRT3* expression, resulting in mitochondrial dysfunction and senescence [36]. Notably, the levels of EV miR-23b-3p and miR-494-3p directly correlate with IPF disease severity. Second, *Sirt3*^−/−^ mice spontaneously develop age-associated fibrosis in a variety of organs, including the lungs, due partly to increased TGF-β1 synthesis arising from acetylation of glycogen synthase kinase B (GSK-β) [31]. *Sirt3*^−/−^ mice are also more susceptible to bleomycin- and asbestos-induced lung fibrosis in association with detrimentally impacted mtOGG1 function, resulting in mtDNA damage in AECs [32] and fibroblasts [33]. Third, overexpression of SIRT3 using either genetic or pharmacologic approaches mitigates oxidant-induced fibroblast profibrotic signaling and AEC mtDNA damage and apoptosis in vitro as well as bleomycin-induced lung fibrosis in vivo [32,33,34,35,37,38] and promotes lung fibrosis resolution in aged mice [35]. Mesenchymal stem cells can diminish diabetic lung fibrosis by augmenting SIRT3 protein expression and SIRT3-mediated stress response [39]. Our group has established a causal role for the recruitment of pro-fibrotic monocyte-derived alveolar macrophages (Mo-AMs) in the development of both bleomycin- and asbestos-induced lung fibrosis, but the role of SIRT3 in modulating recruitment of Mo-AMs is unclear [40,41,42]. Taken together, these findings suggest that SIRT3 is crucial for maintaining mtDNA integrity in the setting of fibrogenic oxidative stress and thereby limits AEC apoptosis and lung fibrosis; however, it is unclear whether transgenic whole body SIRT3 overexpression (*Sirt3^Tg^*) mice are protected against lung fibrosis following asbestos exposure, a non-resolving model of lung fibrosis unlike bleomycin, and if so, whether the protective effects are mediated in part by attenuating lung and AEC mtDNA damage and recruitment of Mo-AMs.

To determine whether overexpression of SIRT3 mitigates asbestos-induced pulmonary fibrosis, we assessed lung fibrosis in *Sirt3^Tg^* as compared to their wild-type (WT) controls. Compared to WT, *Sirt3^Tg^* mice were protected from asbestos-induced pulmonary fibrosis and protection occurred in association with diminished lung mtDNA damage and Mo-AM recruitment. Our in vitro AEC studies also demonstrated that pharmacologic SIRT3 inducers (i.e., resveratrol, viniferin, and honokiol) each diminish oxidant-induced mtDNA damage and, in the case of honokiol, the beneficial effects occurred in a SIRT3-dependent manner. Collectively, these data support an important role for SIRT3-dependent preservation of AEC mtDNA as a novel therapeutic focus for managing patients with IPF and other types of pulmonary fibrosis.

## 2. Results

### 2.1. SIRT3 Overexpression Attenuates Asbestos-Induced Pulmonary Fibrosis

**Figure 1:**
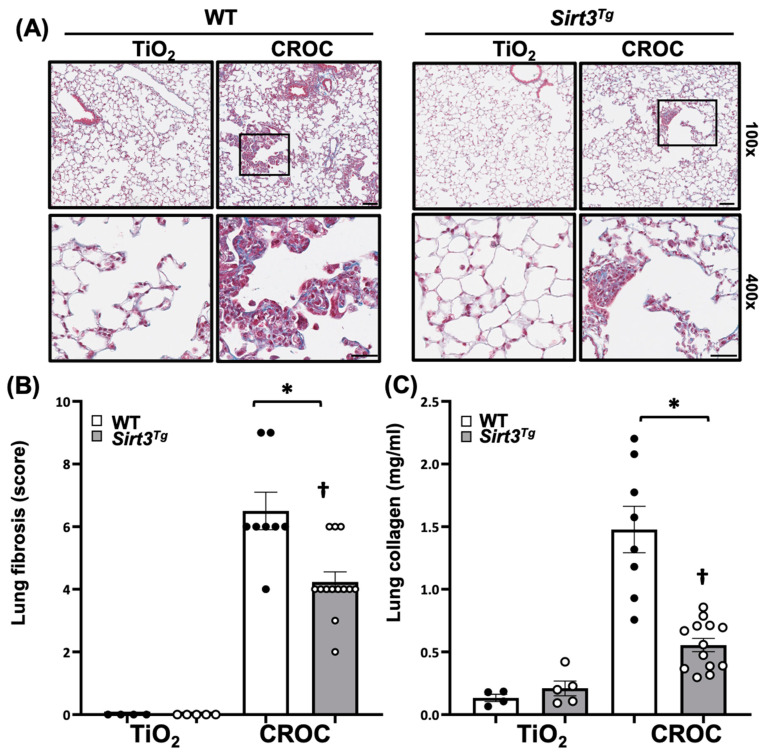
*Sirt3^Tg^* mice are protected against asbestos-induced pulmonary fibrosis. Wild-Type (WT) and *Sirt3^Tg^* mice were harvested 21 days after treatment with a single IT (intratracheal) instillation of crocidolite asbestos or titanium dioxide (TiO_2_) control (100 µg in 50 µL). The extent of fibrosis was determined on day 21 in mouse lung by trichrome staining (**A**), lung fibrosis score (**B**), and collagen levels assessed by a Sircol assay (**C**). *n* = 4–13; * *p* < 0.05 vs. Ti; † *p* < 0.05 vs. WT. Scale bars = 100× = 0.1 mm; 400× = 0.05 mm.

Previous studies have shown that *Sirt3^Tg^* mice, as compared to controls, demonstrate a global ~4-fold increase in SIRT3 expression in all cell types vs. WT mice at baseline and following bleomycin exposure and have reduced bleomycin-induced pulmonary fibrosis [31,33]. However, the murine bleomycin lung fibrosis model is limited by being unlike that seen in patients with IPF, as the histopathology is largely a post-inflammatory fibrosis that typically spontaneously resolves unless multiple bleomycin doses are given [43]. We showed that asbestos-induced lung fibrosis after a single intratracheal (IT) instillation is reproducibly present at 14 d and persists at 60 d [41,42]. We determined whether SIRT3 overexpression is protective in the asbestos lung fibrosis model by using WT (C57Bl6) mice or *Sirt3^Tg^* mice that were exposed to a single IT instillation of crocidolite asbestos (100 μg/50 μL PBS) or non-fibrogenic control particulate (TiO_2_ 100 μg/50 μL PBS). Consistent with our prior studies [12,19,21,41,42], compared to TiO_2_ controls, asbestos fibers increased pulmonary fibrosis at 21d in WT mice as evidenced by histology (trichrome staining—Figure 1A; Supplementary Figure S1), lung fibrosis score assessed by an investigator blinded to the experimental protocol (Figure 1B), and lung collagen levels as assessed by a Sircol assay (Figure 1C). Interestingly, asbestos-induced lung fibrosis was reduced in *Sirt3^Tg^* mice as compared to WT as assessed by histology, lung fibrosis score, and lung collagen levels (Figure 1). Collectively, these studies firmly support that SIRT3 overexpression can mitigate pulmonary fibrosis following asbestos exposure in a manner comparable to that seen following bleomycin exposure.

### 2.2. SIRT3 Overexpression Mitigates Asbestos-Induced mtDNA Damage in Mouse Lungs

**Figure 2:**
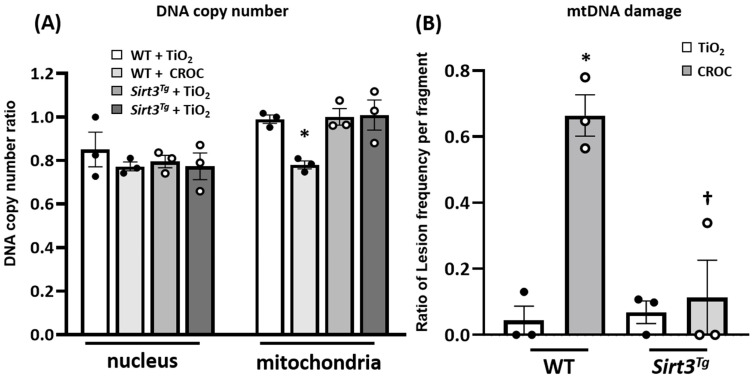
*Sirt3^Tg^* mice exhibit reduced asbestos-induced lung mtDNA damage as compared to WT. Lung tissues from mice in Figure 1 were assessed by a q-PCR assay for lung nuclear and mtDNA copy number (**A**) and lung mtDNA damage (**B**). N = 3; * *p* < 0.05 vs. WT + Ti. † *p* < 0.05 vs. WT + CROC.

To investigate possible mechanistic pathways accounting for the protective effects of SIRT3 in our in vivo asbestos lung fibrosis model, two possibilities were explored. Given that mtDNA damage is important for promoting AEC apoptosis that drives lung aging and fibrosis [5,7,8,9,10,11,12,13,14,16,18,19,20,21] and that SIRT3 overexpression mitigates oxidant (H_2_O_2_ and asbestos)-induced AEC mtDNA damage in vitro [32], we determined whether SIRT3 overexpression reduces asbestos-induced mtDNA damage in lung tissue in vivo. As expected, crocidolite asbestos augments murine lung mtDNA damage at 21 d as compared to TiO_2_ (Figure 2). Notably, *Sirt3^Tg^* murine lungs completely abolished asbestos-associated lung mtDNA damage as compared to WT murine lungs. Taken together, these data suggest that SIRT3 overexpression prevents lung mtDNA damage in asbestos-exposed mice.

### 2.3. SIRT3 Overexpression Diminishes Recruitment of Pro-Fibrotic Mo-AMs

**Figure 3:**
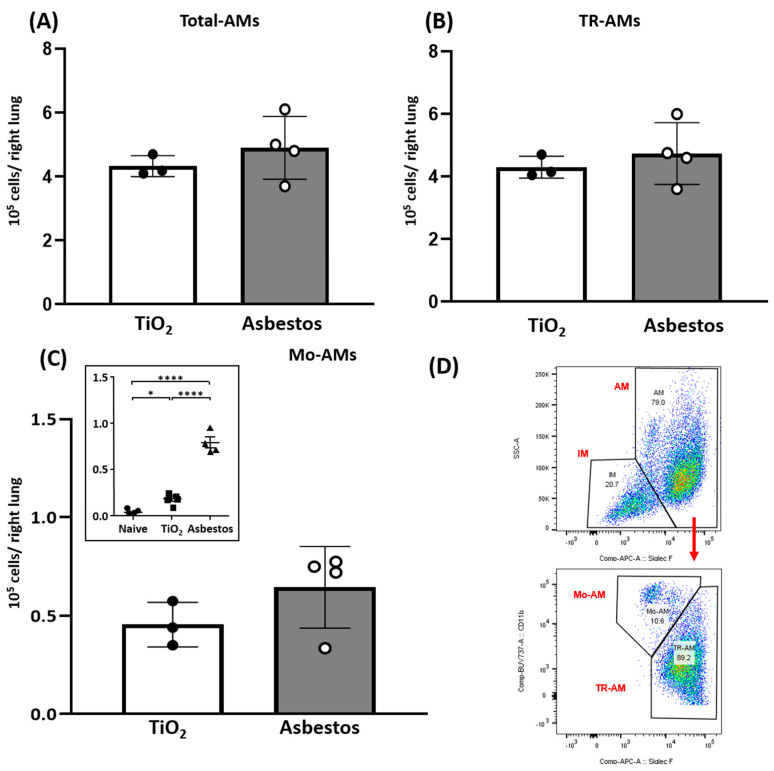
*Sirt3^Tg^* mice do not recruit fibrogenic Mo-AMs to the lung following asbestos exposure. *Sirt3^Tg^* mice were treated with TiO_2_ or crocidolite asbestos (100 µg in 50 µL). Twenty-eight days after treatment, macrophages were purified from lungs and used for flow cytometry to determine recruitment of macrophage subsets, including profibrogenic monocyte-derived alveolar macrophages (Mo-AMs), as described in detail elsewhere (40), Lung cells were assessed for alveolar macrophages ((**A**); AMs), tissue-resident macrophages ((**B**); TR-AMs), and monocyte-derived alveolar macrophages ((**C**); Mo-AMs) from the *Sirt3^Tg^* lungs following TiO_2_ or crocidolite asbestos exposure. For WT mice, Mo-AMs were assessed from the murine lungs following exposure to saline (naïve), TiO_2_, (open bars), or crocidolite asbestos (gray bars) and showed that asbestos induces ~3-fold increase in Mo-AMs as compared to controls ((**C**) Insert). (**D**) Scatterplots showing fate distribution of AMs in mouse lungs, including relative percentage of Mo-AMs and TR-AMs. Please see Supplement Figure 2 for complete gating strategy. Statistics for insert: All data presented as mean ± SEM. One-way ANOVA with Tukey’s test for multiple comparisons; * *p* < 0.05, **** *p* < 0.0001. Insert figure reproduced with the permission of ^©^ERS 2021: European Respiratory Journal 55 (1) 1900646; DOI: 10.1183/12993003.0646-2019 Published 16 January 2020 (Ref #41).

A second mechanism that we explored in our in vivo lung fibrosis model is whether SIRT3 diminishes recruitment of pro-fibrotic Mo-AMs. We have established a causal role for Mo-AM lung recruitment especially into areas of fibrosis [41], accounting for 5–15% of all alveolar macrophages and a gain in their capacity for self-renewal in mediating lung fibrosis following exposure to either bleomycin or asbestos because genetic ablation of Mo-AMs prevented their recruitment into the lungs following epithelial injury and restoration of the Mo-AM population can “rescue” lung fibrosis [40,41,42]. To directly address whether SIRT3 overexpression impacts the recruitment of Mo-AMs, we assessed Mo-AM lung recruitment in a separate group of *Sirt3^Tg^* mice exposed 21 d to either TiO_2_ or asbestos fibers (Supplementary Figure S2). Unlike the ~3-fold increase in Mo-AM lung recruitment that occurs in asbestos-exposed WT mice as compared to TiO_2_ controls that we have previously reported ([41]; Figure 3C Inset), we detected negligible differences in the number of lung Mo-AMs as well as total AMs and tissue-resident macrophages in the *Sirt3^Tg^* mice exposed to either TiO_2_ or asbestos (Figure 3). These data along with our prior studies [40,41,42] suggest that SIRT3 overexpression mitigates recruitment of the pro-fibrotic Mo-AMs into murine lungs following asbestos exposure.

### 2.4. Pharmacologic Agents That Augment SIRT3 Levels Diminish Oxidant-Induced AEC mtDNA Damage

**Figure 4:**
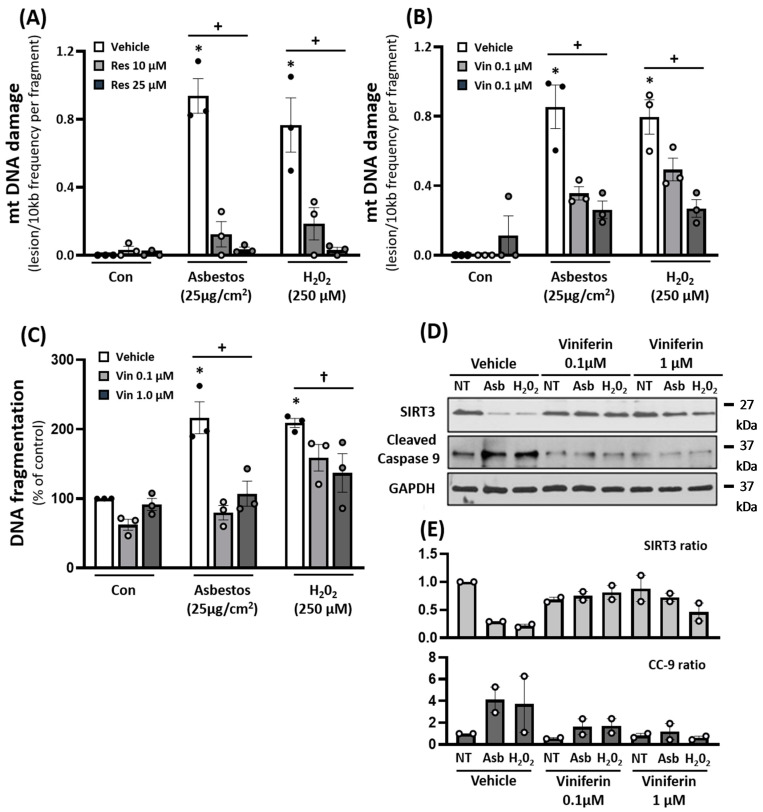
The SIRT3 agonists resveratrol and viniferin reduced oxidant-induced mtDNA damage, preserved SIRT3 expression, and reduced apoptosis in human AEC, as assessed by DNA fragmentation and CC-9 expression. A549 cells were pre-treated with resveratrol (10–25 µM) or viniferin (0.1–1 µM) for 6 h then exposed to either amosite asbestos (25 µg/cm^2^) or H_2_O_2_, (250 µM), and 24 h later the cells were harvested for mtDNA damage, DNA fragmentation, or Western blotting. Resveratrol (**A**) and viniferin (**B**) mitigate oxidant-induced mtDNA damage. Viniferin ameliorates oxidant-induced apoptosis as assessed by DNA fragmentation (**C**) and cleaved caspase-9 (**D**,**E**) and preserved SIRT3 expression (**D**,**E**). For (**A**–**D**), *n* = 3, for E, *n* = 2; * *p* < 0.05 vs. vehicle, + *p* < 0.05 vs. vehicle, † *p* = 0.07 vs. vehicle.

Small molecule sirtuin stimulators that augment SIRT3 (i.e., resveratrol, viniferin, and honokiol) can attenuate bleomycin-induced fibrosis in the lungs, skin, heart, and kidneys [37,44,45,46,47,48,49]. However, the role of these agents in modulating oxidant-induced AEC mitochondrial function and apoptosis has not been studied. To address this information gap, we performed in vitro studies using cultured AEC cell lines (i.e., A549 and MLE12 cells). Compared to vehicle control (DMSO 0.1%), resveratrol (10–25 µM) abolished oxidant (amosite asbestos and H_2_O_2_)-induced A549 cell mtDNA damage (Figure 4A). In a similar manner, viniferin, a resveratrol derivative, also reduced oxidant-induced A549 cell mtDNA damage, and the protective effects occurred in a viniferin dose-dependent manner (Figure 4B). Further, the beneficial effects of viniferin were associated with augmented SIRT3 expression and attenuation of intrinsic AEC apoptosis as assessed by assays of DNA fragmentation and cleaved caspase-9 (CC-9) (Figure 4C,D).

**Figure 5:**
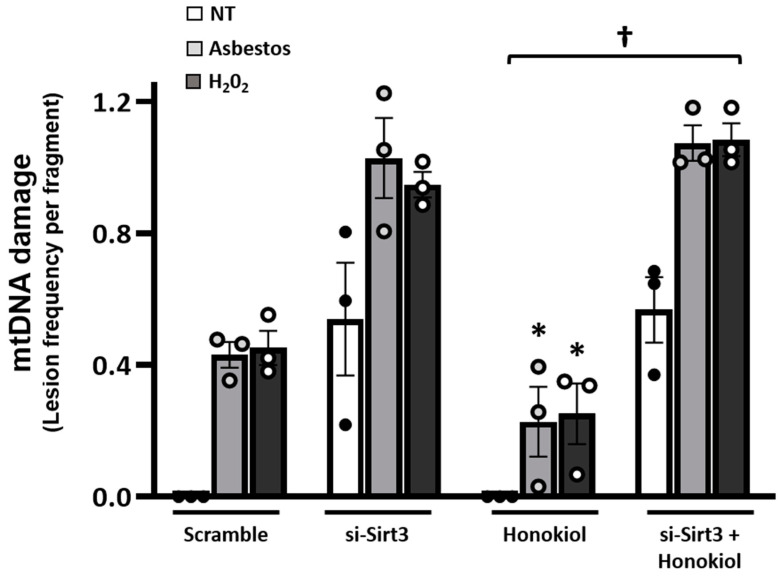
Honokiol, a specific SIRT3 inducer, attenuates MLE-12 cell oxidant-induced mtDNA damage in a SIRT3-dependent manner. Forty-eight hours after transfection with scramble or Sirt3 siRNA, MLE cells were treated for 6 h with 10 µM honokiol, followed by 24-h treatment with amosite asbestos @ 25 µg/cm^2^ or 250 µM H_2_O_2_. mtDNA damage expressed as lesion frequency per DNA fragment. All *n* = 3; * *p* = 0.07 vs. scramble + Asb or H_2_O_2_; † *p* < 0.05 vs. honokiol + Asb, or honokiol + H_2_O_2_.

Because non-SIRT3-dependent mechanisms have been implicated in mediating the protective effects of resveratrol and possibly its derivatives, we explored the protective effects of honokiol, a specific SIRT3-dependent agent [28,37,44,45,46,47,48,49]. Honokiol preserves mitochondrial function and protects the heart from doxorubicin-induced cardiomyopathy in mice in a SIRT3-dependent manner [49]. Our group showed that hexafluoro, a more lipophilic derivative of honokiol, augments SIRT3 levels in the skin and lungs of mice and mitigates bleomycin-induced lung and skin fibrosis [37]. Herein, we show that, compared to vehicle control (DMSO 0.1%), honokiol (10–25 µM) attenuates oxidant (amosite asbestos and H_2_O_2_)-induced MLE12 cell mtDNA damage by ~50% and trended toward statistical significance (*n*= 3; *p* = 0.07) (Figure 5). Notably, the protective effects of honokiol were completely abolished in the presence of siRNA SIRT3, which completely blocks MLE SIRT3 protein expression (Supplementary Figure S3), suggesting that protection occurred in a SIRT3-dependent manner (*p* < 0.05: honokiol + asbestos or H_2_O_2_ v. siSIRT3 + honokiol + asbestos or H_2_O_2_; *n* = 3; Figure 5).

## 3. Discussion

Accumulating evidence has firmly implicated a key role for SIRT3 deficiency in the pathobiology of IPF as well as the bleomycin murine model of lung fibrosis, but the detailed molecular mechanisms involved are not fully understood, nor is it known whether augmenting SIRT3 in mice following exposure to asbestos is protective [32,33,34,35,36,37,38,39]. In this study we addressed these two important gaps in our understanding of the role of SIRT3 in modulating pulmonary fibrosis by showing that *Sirt3^Tg^* mice are protected from asbestos-induced pulmonary fibrosis and that lung protection is associated with reduced levels of lung mtDNA damage and fibrogenic Mo-AM recruitment. Furthermore, pharmacologic SIRT3 inducers (resveratrol, viniferin, and honokiol) mitigate oxidant-induced AEC mtDNA damage and silencing SIRT3 blocks the beneficial effects of honokiol, suggesting that protection occurs in a SIRT3-dependent manner.

A major finding in this study is that *Sirt3^Tg^* mice, which globally overexpress SIRT3, have reduced pulmonary fibrosis as compared to WT mice 21 d following asbestos exposure (Figure 1). The protective effects observed herein with SIRT3 overexpression are comparable to the effects reported following bleomycin exposure [33]. A unique feature of our study involved use of the murine asbestos lung fibrosis model, which unlike bleomycin, does not spontaneously resolve [19,42,43]. Although there are an abundant number of pharmacologic agents that can reduce lung fibrosis when administered before or at the time of bleomycin exposure, the most common murine lung fibrosis model, there are only a few agents that maintain their beneficial effects when initiated following exposure to bleomycin, which is another limitation of the bleomycin lung fibrosis model [43]. Our group has been using the asbestos lung fibrosis model because it reproducibly causes lung fibrosis as early as 14 d, persists at 60 d (the longest time point that we have studied), and enables post-exposure studies for assessing innovative treatments [12,19,21,41,42]. Given the importance of sphingosine kinase 1 (SPHK1) in promoting pulmonary fibrosis, we recently reported the first asbestos post-exposure study demonstrating that PF543, a specific SPHK1 inhibitor, can attenuate lung fibrosis when initiated 1 month following asbestos exposure [42]. Further, the protective effects of PF543 occurred in association with reduction in lung expression of profibrotic markers, lung mtDNA damage, and fibrogenic monocyte recruitment, all of which parallel findings in the present study [42]. Although PF543-induced alterations in SIRT3 levels are unknown, PF543 can attenuate mtROS production [50] in a manner similar to that of SIRT3 [24,25,26,27,28,29,30].

The precise mechanism(s) by which SIRT3 overexpression mitigates lung fibrosis is not fully established from these studies; however, we investigated two possibilities: (i) reducing mtDNA damage and (ii) ameliorating lung recruitment of fibrogenic Mo-AMs. First, we investigated whether SIRT3 can attenuate mtDNA damage in asbestos-exposed murine lungs as well as cultured AECs because mtDNA damage is prominently implicated in mediating AEC mitochondria-regulated (intrinsic) apoptosis that can drive aging and lung fibrosis [5,7,8,9,10,11,12,13,14,16,18,19,20,21]. We showed that *Sirt3^Tg^* mice, unlike WT controls, have no detectable lung mtDNA damage following asbestos exposure (Figure 2). Further, our AEC in vitro studies demonstrated that three agents that are well-known for augmenting SIRT3 expression [44,45,46,47,48,49] can each block oxidant (asbestos or H_2_O_2_)-induced AEC mtDNA damage (Figure 4 and Figure 5). The protective effects of each pharmacologic agent in diminishing oxidant-induced AEC mtDNA damage parallel our findings using a genetic approach to augment AEC SIRT3 expression [32]. Our observation that honokiol protection against oxidant-induced AEC mtDNA damage occurs in a SIRT3-dependent manner concurs with prior studies showing that honokiol’s protective effects are dependent upon SIRT3 in ameliorating murine bleomycin-induced pulmonary fibrosis [37], doxorubicin-induced cardiomyopathy [49], diabetes-induced myocardial dysfunction [51], and radiation-induced brain injury [52]. Although the mechanism by which SIRT3 reduces mtDNA damage was not explored in this study, prior studies have established that SIRT3 can reduce mtROS levels and enhance mtDNA repair in a variety of cell types [24,25,26,27,28,29,32,33,53,54]. Bleomycin-induced lung fibrosis reduces lung SIRT3 and OGG1 expression in WT but not *Sirt3^Tg^* mice [33]. SIRT3 is crucial for deacetylating key mitochondrial proteins, thereby preserving their antioxidant (i.e., MnSOD) and mtDNA repair (i.e., mtOGG1) functions. Our findings herein add to the accumulating evidence implicating a role for mtDNA damage in mediating lung fibrosis in humans with IPF as well as murine models of lung fibrosis [5,9,10,11,12,13,14,16,18,19,20,21,32,42]. Notably, *MCAT* mice that globally overexpress mitochondria-targeted human catalase have reduced asbestos- and bleomycin-induced lung fibrosis that occurs in association with reduced levels of AEC mitochondrial ROS production and AEC and lung mtDNA damage [12]. Further, we demonstrated that asbestos- and bleomycin-induced pulmonary fibrosis is augmented in *Ogg1*^−/−^ mice but attenuated in mice globally overexpressing the human mitochondria-targeted OGG1 subunit 1-alpha transgene (*mtOgg1^tg^*) with parallel up- and down-regulation in AT2 cell mtDNA damage and apoptosis, respectively [19,21]. Extracellular MtDNA release into the plasma or BALF, which was not explored in this study, can arise from fibroblasts exposed to TGFβ or AECs undergoing apoptosis and is directly associated with IPF mortality [22,23]. Collectively, these data suggest that SIRT3 overexpression can diminish lung and AEC mtDNA damage following exposure to agents that induce lung fibrosis, such as asbestos or bleomycin.

A second mechanism that we explored was whether SIRT3 overexpression mitigates lung fibrosis by reducing the recruitment of profibrotic Mo-AMs. Indeed, we observed similar levels of lung Mo-AMs in the *Sirt3^Tg^* mice following exposure to either asbestos or non-fibrogenic particulate controls (TiO_2_) (Figure 3). These findings are unlike the nearly 3-fold increase in Mo-AMs recruited into the fibrotic lungs of asbestos-exposed WT mice after 14–60 d (Figure 3C, inset; [41,42]). These data in the *Sirt3^Tg^* mice suggest that SIRT3 overexpression attenuates lung Mo-AM recruitment following asbestos exposure and add to the accumulating evidence implicating Mo-AMs in the pathobiology of pulmonary fibrosis in mice as well as in humans with IPF [40,41,42,55,56,57,58].

Although the causal role of lung recruitment of Mo-AMs in mediating lung fibrosis is established, the precise molecular mechanisms involved are unclear and an area of ongoing study by our group and others [35,40,41,42,55,56,57,58]. Using the asbestos lung fibrosis model with a combination of confocal microscopy and single-cell RNA sequencing, our group reported that Mo-AMs are recruited to fibrotic areas of the lungs, engulf asbestos fibers, and provide a connection between injured alveolar epithelium and activated fibroblasts by localizing in spatially restricted pro-fibrotic niches [41]. Further, our transcriptomic studies identified two factors released from Mo-AMs that are potentially important for promoting lung fibrosis: (i) platelet-derived growth factor A, which can drive fibroblast proliferation and activation, and (ii) macrophage colony-stimulating factor receptor signaling, which appears crucial for Mo-AM self-maintenance and persistence. Others have recently reported a beneficial role for Mo-AM-derived apolipoprotein E (ApoE) for the resolution of bleomycin-induced pulmonary fibrosis [57]. Collectively, these studies implicate Mo-AMs in the pathobiology of pulmonary fibrosis, but additional studies are warranted to better understand the mechanisms involved as well as how modulating aspects of Mo-AM biology may serve as a novel management target in patients with IPF.

SIRT3 may attenuate pro-fibrotic signaling in other cells within these fibrosis-promoting distal lung niches. Consistent with this notion is that SIRT3 deficiency is evident in the AECs and fibroblasts of patients with IPF as well as aged mice [32,33,35]. Notably, a recent study showed that airway delivery of a *Sirt3* overexpression cDNA promotes resolution of lung fibrosis in aged mice in part by working along with macrophage-derived paracrine secreted products in activating the forkhead box (FOX) transcription factor FoxO3a in fibroblasts, which subsequently augments fibroblast pro-apoptotic Bcl2 family member expression and apoptosis [35]. It will be of considerable interest to determine whether conditional SIRT3 overexpression in AECs, fibroblasts, and/or macrophages is required for mitigating pulmonary fibrosis.

There are some limitations from this study. First, although we have identified at least two mechanisms that may account for the protective effects of SIRT3 overexpression in mitigating lung fibrosis following asbestos exposure, further studies are necessary for elucidating the precise molecular mechanisms involved as well as how SIRT3 prevents lung mtDNA damage and recruitment of lung Mo-AMs. Second, we have focused on AECs in these studies while, as noted above, others have investigated how SIRT3 deficiency impacts fibroblast biology necessary for promoting lung fibrosis [33,35,36]. A better understanding of the causal relationship between SIRT3 expression among the multiple key lung cell types mediating pulmonary fibrosis, especially AECs, fibroblasts, and Mo-AMs, will be important along with identifying crucial cross-talk signaling between cell types for promoting epithelial mesenchymal transition and pulmonary fibrosis. Finally, it will be of interest to determine whether SIRT3 impacts mtDNA release from various cells involved in lung fibrosis and how mtDNA signaling may alter lung repair and immune responses in health, disease/fibrosis, and aging. Finally, we use transformed AEC cell lines (MLE-12 and A549) for these studies rather than primary isolated AT2 cells. However, our previous studies showing that these AEC lines show similar changes in oxidant-induced SIRT3 expression, mitochondrial dysfunction, and apoptosis, as we see in primary murine and human AT2 cells, suggest that this does not alter the major conclusions of the present study while enabling our transfection studies, which are not feasible in primary AT2 cells because they lose their phenotype after 48 h in culture [12,21,32]. Despite these limitations, our findings clearly suggest that SIRT3 overexpression can attenuate lung fibrosis following asbestos exposure in association with reduced lung mtDNA damage and recruitment of Mo-AMs.

In summary, using the murine asbestos lung fibrosis model that does not spontaneously resolve, we demonstrated that *Sirt3^Tg^* attenuates pulmonary fibrosis. Further, we showed that the protective effects of SIRT3 overexpression are associated with reduced lung mtDNA damage and Mo-AM recruitment. Moreover, we found that pharmacologic SIRT3 overexpression can abolish oxidant-induced AEC mtDNA damage and that the protective effects of honokiol were blocked in the presence of silencing of SIRT3, which suggests a crucial role for SIRT3. Collectively, these findings support the anti-aging role of SIRT3 [59] in mitigating pulmonary fibrosis in part by maintaining AT2 cell mtDNA integrity that may be necessary for preserving the "stem" cell role of AT2 cells in the setting of fibrogenic stimuli (i.e., asbestos). Given the important role of AEC mtDNA damage in promoting mitochondria-regulated apoptosis and subsequent pulmonary fibrosis, we reason that strategies aimed at augmenting lung SIRT3 levels are an innovative therapeutic target for managing patients with IPF and other forms of lung fibrosis.

## 4. Methods

### 4.1. Reagents

The inert control particle, titanium dioxide (TiO_2_), 30% hydrogen peroxide (H_2_O_2_) solution, viniferin, resveratrol, and honokiol were purchased from EMD-Millipore/Sigma (St Louis, MO, USA). Crocidolite and amosite asbestos fibers used in the study are Union International Contre le Cancer (UICC) reference standards, kindly supplied by Dr. Andy Ghio, U.S. Environmental Protection Agency [20]. Antibodies for flow Western blotting and flow cytometry were purchased from commercial vendors and previously verified ([42] and Supplementary Table S1).

### 4.2. Animals

All animal studies in this manuscript were approved by the Northwestern University and Jesse Brown VA Medical Center Animal Use and Care Committees (IACUCs; NU IACUC protocol # IS0007912 and JBVAMC protocol # 16-04). Male and female 8- to 10-week-old *C57Bl/6J* wild-type (WT; Jax 00664) and *Sirt3^Tg^* mice were kindly provided by David R. Gius (Northwestern University).

### 4.3. Asbestos Instillation into Mice

IT instillation of TiO_2_ or crocidolite asbestos was performed as previously described [12,19,21,41,42]. Eight- to 10-week-old male or female WT (*C57Bl/6J*) or *Sirt3^Tg^* mice were orally intubated with a 20-gauge angiocatheter (BD Biosciences, Sandy, UT, USA) with suspensions of TiO_2_ or crocidolite asbestos. One hundred micrograms of each were instilled in 2 equal 25 µL aliquots, given 2 min apart. After each aliquot, the mice were placed in the right and left decubitus position for 10–15 s.

### 4.4. Titanium Dioxide and Asbestos Preparation for Instillation into Mice

TiO_2_ or crocidolite asbestos were prepared as described previously [12,19,21,42]. TiO_2_ particles and crocidolite fibers were suspended in phosphate buffered saline (PBS) and 15 mM HEPES at 2 mg/mL. Crocidolite was sonicated for 8 min at 40% power to disrupt fiber clumps (Sonicator: Branson, Danbury, CT, USA). Both suspensions were autoclave-sterilized before use.

### 4.5. Lung Harvest and Histology

Lungs were harvested 21 to 28 days after IT instillation of asbestos or TiO_2_ control as described previously [12,19,21,42]. Briefly, a 20-gauge cannula was sutured into the trachea, and the right lung was ligated at the hilum after removal of the left lung, which was saved for biochemical collagen determination, and inflated to 15cm H_2_O with 10% formalin. The right lung was then dehydrated, embedded in paraffin, and then serial 5 µM sections were stained with hematoxylin and eosin (H&E) and Masson’s Trichrome.

### 4.6. Lung Collagen Detection

For soluble collagen determination, the left lung was homogenized in 0.5N acetic acid using a polytron (Kinematica, Bohemia, NY, USA) followed by Dounce homogenization and clearing by centrifugation. Equal volumes of cleared homogenate were subject to the Sircol assay for soluble collagen based on a modified picrosirius red (EMD-Millipore/Sigma, St. Louis, MO, USA) collagen precipitation assay, which, as described previously [42], parallels protein expression of lung type 1 collagen levels [19].

### 4.7. Fibrosis Scoring System

Lung sections were assessed for fibrosis scores as previously described by a pulmonary pathologist, blinded to our experimental protocol [12,19,21,42]. Lungs were scored on the severity of fibrosis from 0 (no fibrosis) to 4 (severe fibrosis) and also on the extent of involvement, which was quantified on a scale of 1 (occasional duct and bronchiole involvement) to 3 (more than half of the alveolar ducts and respiratory bronchioles involved). Severity (0–4) and extent (1–3) were multiplied together to yield the fibrosis score.

### 4.8. Quantitative mtDNA Damage Assay via PCR

Nuclear and mtDNA damage were assessed as described previously [9,12,21,42]. Genomic DNA from paraffin-embedded lungs or cultured cells were extracted using the Qiagen Genomic Tip 20/G and Qiagen DNA Buffer Set (Qiagen, Gaithersburg, MD, USA) according to the manufacturer’s protocol. Ex-Taq (Takara, Mountain View, CA, USA) was used for PCR with specific primers to amplify a mitochondrial gene, both in short and long-forms and also nuclear beta globin DNA [9,12,42]. We used PicoGreen for DNA quantification (Thermo-Fisher/Invitrogen, Waltham, MA, USA) using the FL600 microplate fluorescence reader (Thermo-Fisher, Pittsburgh, PA, USA), with excitation and emission wavelengths of 485 and 530 nm, respectively. Mitochondria short-fragment data were used to normalize fluorescence from the mitochondrial long fragment. The number of mitochondrial lesions was calculated by the equation: *D* = (1 − 2^–(Δlong-Δshort)^) × 10,000 (bp)/size of the long fragment (bp).

### 4.9. DNA Fragmentation

DNA fragmentation (as a measure of apoptosis) was measured using a histone-associated DNA fragmentation ELISA kit (Cell Death Detection ELISA^PLUS^, EMD-Millipore/Sigma), which measures mono- and oligo-nucleosomes, according to the manufacturer’s instructions. [9,12,21].

### 4.10. Western Blotting

Immunoblot analysis was performed as we have previously described [9,19,21,42]. Cells were collected and lysed in protein lysis buffer (Cell Signaling, Danvers, MA, USA) with protein and phosphatase inhibitors (Thermo-Fisher/Pierce, Rockford, IL, USA) and cleared by centrifugation. Protein concentration of the supernatant was quantified and boiled in Laemmli sample buffer for 5 min. Cell lysate (20 µg) was separated on gradient or 10% PAGE gels, transferred to nitrocellulose, and blocked with 5% BSA before overnight incubation with primary antibodies, diluted at 1/1000 at 4 °C, followed by HRP conjugated secondary antibody incubation, diluted 1/2000, for 1 h at room temperature (Supplementary Table S1). An ECL chemiluminescence kit (GE Healthcare/Sigma, St. Louis, MO, USA) was used for visualization on X-ray film, and bands were quantified (as integrated density) using Image J software (NIH, Bethesda, MD, USA).

### 4.11. Cell Culture

The human and mouse lung alveolar epithelial type II-like cell lines (A549 and MLE-12, respectively) were purchased from the American Type Culture Collection (Manassas, VA, USA) and maintained in DMEM (Thermo-Fisher-Invitrogen, Grand Island, NY, USA) with 2 mM L-glutamine supplemented with 10% fetal bovine serum, penicillin (100 units/mL), and streptomycin (100 µg/mL). Cells were plated in 6-well plates or 100 mm dishes and grown to 70% confluence for pre-treatment with DMSO in media (control, EMD-Millipore/Sigma), honokiol (10 µM in media, 6 h, EMD-Millipore/Sigma), or resveratrol (10–25 µM in media, 6 h, EMD-Millipore/Sigma), followed by 24 h treatment with amosite asbestos, 25 µg/cm^2^, or 250 µM H_2_O_2_.

### 4.12. Sirt3 Gene Silencing

For *Sirt3* gene silencing experiments, MLE cells were transiently transfected with Sirt3- specific small interfering RNA (Cat. # 132001, Assay ID mss227072, target sequence GCC TCT ACA GCA ACC TTC AGC AGT A) or scramble siRNA control (Silencer Select negative control siRNA, Cat. # 4390844), using Lipofectamine RNAiMax for 48 h, and then used as we previously described [32]. All siRNA and transfection reagents were purchased from Fisher-Thermo/Invitrogen (Waltham, MA, USA).

### 4.13. Cell Isolation, Staining, Flow for Mo-AMs

Cell isolation from lung tissues, flow staining, and analysis were all performed as previously described [40,41,42] and outlined in Supplementary Figure S2. Briefly, after mouse euthanasia and lung perfusion through the right ventricle with 10mL HBSS, mouse lungs were treated with collagenase D (2 mg/mL, EMD-Millipore/Sigma) and DNAse1 (EMD-Millipore/Sigma), previously dissolved in HBSS with Ca^2+^ and Mg^2+^, using a 30 ga syringe. Lungs were chopped with scissors, transferred to a C-tube (Miltenyi, Auburn, CA) to generate a single-cell suspension, and processed in a GentleMACS dissociator (Miltenyi) to generate a single-cell suspension, which was then filtered through a 40 μM nylon strainer. CD 45+ cells were subject to positive selection as follows: the cell suspension was incubated with CD 45+ microbeads, then collected into a MultiMACS Cell 24 separator (Miltenyi). After staining with acridine orange/propidium iodide (AOPI, Nexelcom, Lawrence, MA, USA) reagent, cells were counted on a Cellometer K2 automatic cell counter (Nexelcom). After staining with fixable visibility dye (eFluor506, eBioscience, San Diego, CA, USA) and FcBlock (BD Biosciences, San Jose, CA, USA), cells were stained with the following antibody panel: MHCII:2G9 (BUV395, BD, Cat. # 743876), Ly6C:HK1.4 (eFluor450, eBioscience, Cat. # 48-5932-82), CD45: 30-F11 (fluorescein isothiocyanate (FITC), eBioscience, Cat. # 11-0451-82), CD64 X54-5/7.1 (phycoerythrin (PE), BioLegend, San Diego, CA, USA, Cat. # 558455), Siglec F: E50-2440 (PECF594, BioLegend, Cat. # 562757), CD11c: HL3 (PECy7, BD, Cat. # 561022), CD24: 1/69 (allophycocyanin (APC), eBioscience, Cat. # 17-0242-82), CD11b: M1/70 (APC Cy7, BioLegend, Cat. # 101216), Ly6G:1A8 (Alexa 700, BD Cat. # 56-9668-82), and NK1.1: PK (Alexa 700, BD, Cat. # 560515). BD CompBeads and Arc beads (Thermo-Fisher/Invitrogen), Carlsbad, CA, USA) were used for preparation of single-color controls. Flow cytometry and data acquisition were performed at the Northwestern University Robert H Lurie Comprehensive Cancer Center Flow Cytometry Core (Chicago, IL, USA), using a custom-designed BD FACS Symphony instrument with the BD FACS Diva software (BD). We used FlowJo software (TreeStar; www.flowjo.com, assessed on June 6, 2019) for compensation and data analysis. As described in detail elsewhere [40,41,42], we used a sequential gating strategy and obtained the cell count for each gate by multiplying the live cell percentages from the Cellometer automatic cell counts by the percentage of the cells in the live/singlets gate.

### 4.14. Statistical Analysis

For the in vivo lung fibrosis studies (fibrosis score and Sircol), 4–13 mice were used per group. For the in vivo lung mtDNA and Mo-AM studies, 3–4 animals per group were used. The results of each experimental in vitro condition were determined from the mean of duplicate or triplicate trials. Data were expressed as the mean ± SEM (*n* = 3 unless otherwise stated). An independent sample two-tailed Student’s t-test was used to assess the significance between 2 matched groups. Analysis of variance was used when comparing more than 2 groups to a single control; differences between 2 groups within the set were analyzed by a Fisher’s protected least significant differences test. Probability values < 0.05 were considered significant.

## Data Availability

Not applicable.

## Supplementary Materials

The following are available online at https://www.mdpi.com/article/10.3390/ijms22136856/s1.

## Abbreviations

SIRT3	sirtuin-3
*Sirt3^Tg^*	transgenic whole body SIRT3 overexpression
WT	wild-type
AEC	alveolar epithelial cell
AT2	alveolar type II cell
ROS	reactive oxygen species
mtDNA	mitochondrial DNA
mtOGG1	mitochondrial 8-oxoguanine-DNA glycosylase 1
OGG1	8-oxyguanine DNA glycosylase
BER	base excision repair
BALF	bronchoalveolar lavage fluid
MnSOD	manganese superoxide dismutase
EVs	extracellular vesicles
ER	endoplasmic reticulum
H&E	hematoxylin and eosin
HLF	human lung fibroblasts
*mtOgg1^tg^*	human mitochondria-targeted OGG1 subunit 1-alpha transgene
IPF	idiopathic pulmonary fibrosis
IP	intra-peritoneal
IT	intra-tracheal
MCAT	mitochondrial catalase enforced-expression
α-SMA	alpha-smooth muscle actin
TGFβ	transforming growth factor-beta
