# Supplementary material for: SIRT3 Overexpression Ameliorates Asbestos-Induced Pulmonary Fibrosis, mt-DNA Damage, and Lung Fibrogenic Monocyte Recruitment
Source: Int J Mol Sci. 2021 Jun 25;22(13):6856. doi: 10.3390/ijms22136856 (PMC8268084; doi:10.3390/ijms22136856)

Supplementary Figure S1. ***Sirt3^{Tg}*** **mice are protected against asbestos-induced pulmonary fibrosis.** Whole lung images. Fibrotic bronchoalveolar duct junction area in Figure 1 (Black square). Airway wall collagen (red circles). Magnification = 1.25X

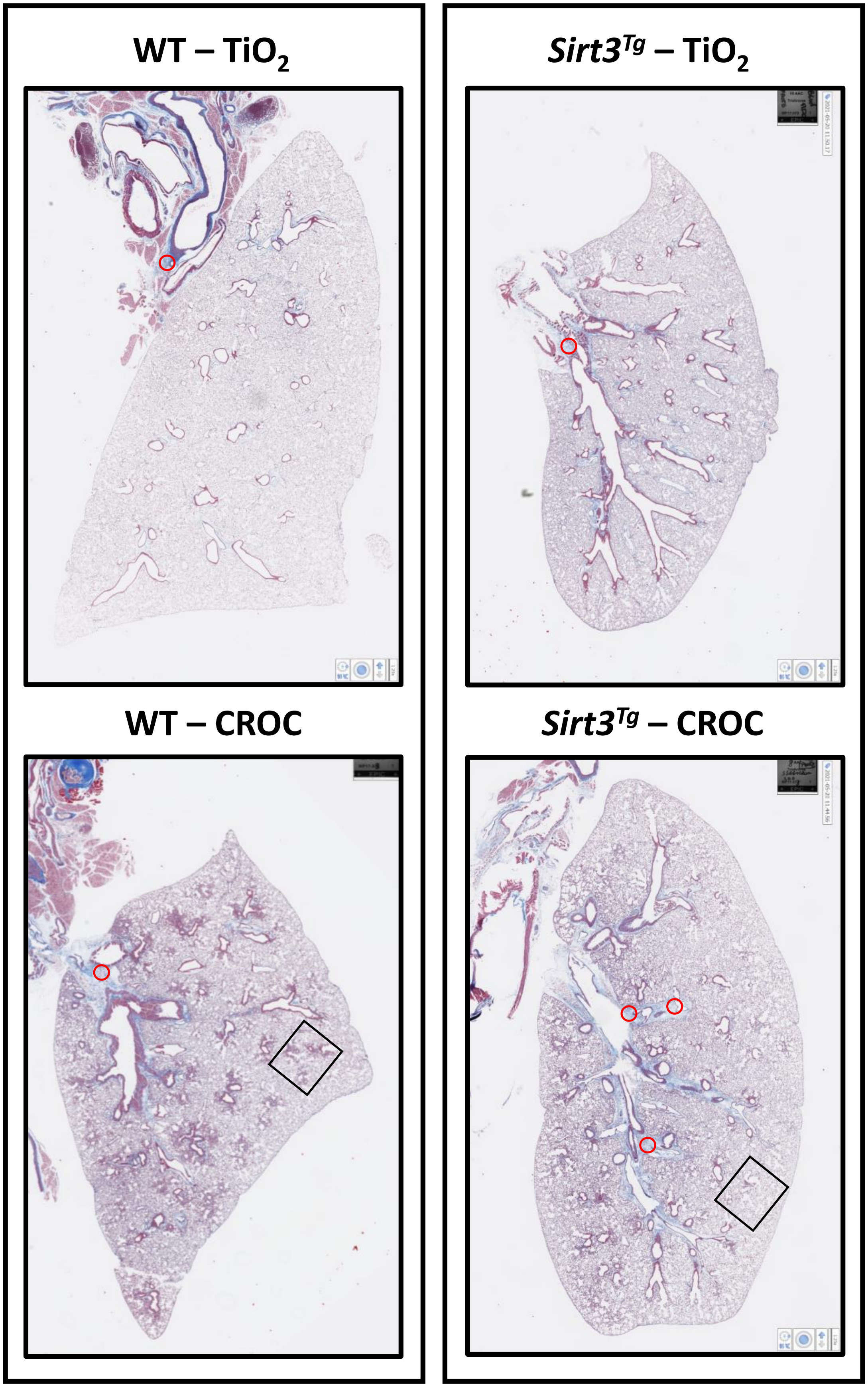

Supplementary Figure S2.

(A) Experimental Design.

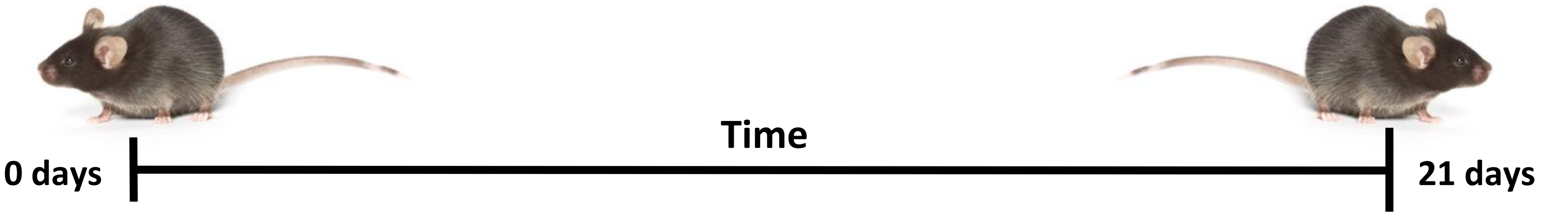

Experimental groups

Group 1:

1.WT (*C57Bl/6J*) + TiO_2 100 μg or UICC Crocidolite asbestos 100 μg.

2.*Sirt3^{Tg}* + TiO_2 100 μg or UICC Crocidolite asbestos, 100 μg.

Lung 1 { **Sircol assay/ lung collagen.**

Lung 2 { **Histology/ lung fibrosis score/ mitochondrial DNA damage q-PCR.**

Group 2:

1.WT (*C57Bl/6J*) + TiO_2 100 μg or UICC Crocidolite asbestos 100 μg.

2.*Sirt3^{Tg}* + TiO_2 100 μg or UICC Crocidolite asbestos, 100 μg.

Lungs 1 & 2 { **Mo-AM Flow Cytometry**

(B) Flow gating strategy. Twenty-one days after exposure to 100ug TiO_2 or Crocidolite asbestos, lungs were harvested from WT (C57Bl/6J) or Sirt3Tg mice, digested the lung enzymatically to generate a single-cell suspension, and stained with the panel of antibodies *described in 4.13 of the Methods section of this manuscript*. After the exclusion of doublets and debris (Panel **B**), live immune cells were identified by CD45 and live/dead staining (Panel **C** and **D**). A sequential gating strategy was used to identify populations expressing specific markers: neutrophils (Ly6G+), NK cells (NK1.4+), eosinophils (CD11b+ CD64- Siglec F+), interstitial macrophages (CD64+ Siglec F-), and alveolar macrophages (CD64+ Siglec F+) (Panel **E**-**J**). Alveolar macrophages (AMs) were categorized as tissue-resident AMs (TR-AM) and monocyte-derived AMs (Mo-AM), based on the expression levels of CD11b and Siglec F: TR-AM (Siglec F^{high} $CD11b^{low}$) and Mo-AM (Siglec F^{low} $CD11b^{high}$) (Panel **K**). Please see refs 41 and 42 for details.

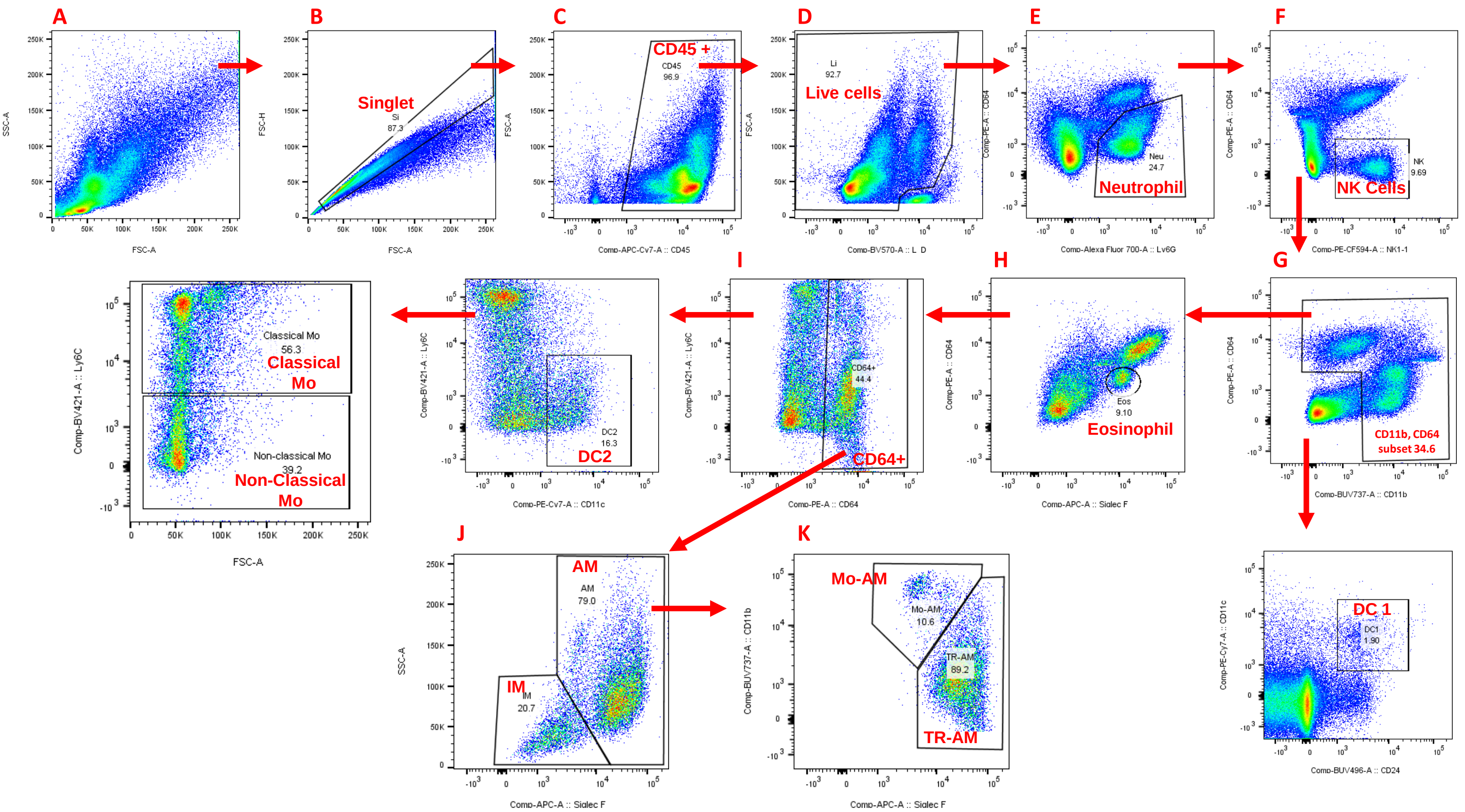

Supplementary Figure S3. ***Sirt3 protein expression is silenced MLE cells treated with Sirt3 siRNA*.** SIRT3 ~ 28 kDa; GAPDH ~37kDa (Panel **A**), N=3.

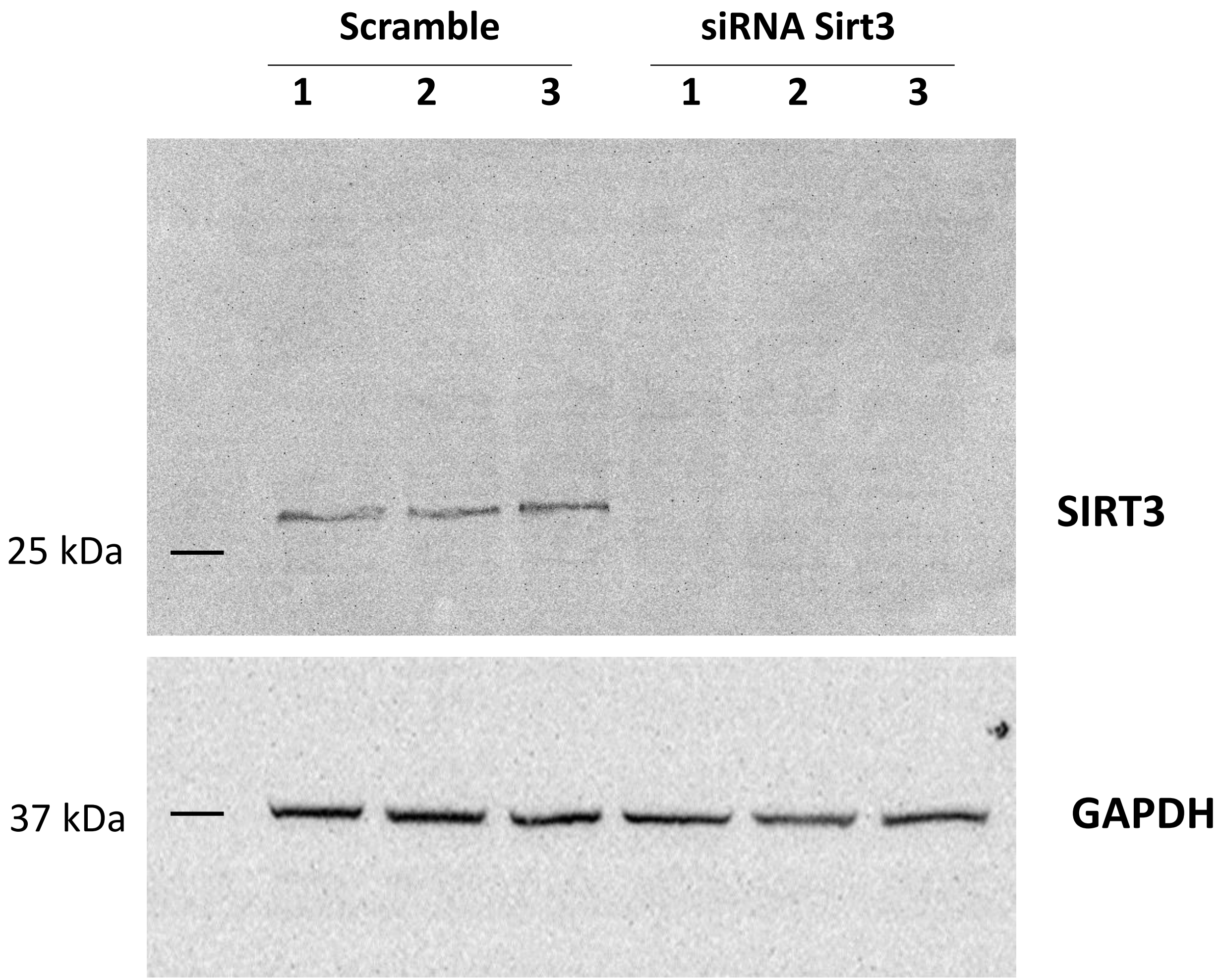

Supplement: Supplementary file 1 [file ijms-22-06856-s001.zip › ijms-1190396-supplementary-checked.pdf]
